# Assessments of the ocular surface and meibomian gland morphology in patients with treatment-naive acne vulgaris

**DOI:** 10.5935/0004-2749.20230025

**Published:** 2022-02-18

**Authors:** Semra Koca, Ayşe Yeşim Oral

**Affiliations:** 1 Department of Ophthalmology, Faculty of Medicine, Afyonkarahisar Health Sciences University Afyonkarahisar, Turkey

**Keywords:** Acne vulgaris/complication, Acne vulgaris/drug therapy, Isotretinoin/therapeutic use, Eyelid, Conjunctiva/pathology, Dry eye syndrome, Meibomian gland, Diagnostic technique, ophthalmological, Questionnaire, Acne vulgar/complicação, Acne vulgaris/tratamento farmacológico, Isotretinoína/uso terapêutico, Pálpebra, Túnica conjuntiva/patologia, Síndrome do olho seco, Glândula tarsal, Técnica de diagnóstico oftalmológico, Questionário

## Abstract

**Purpose:**

To investigate ocular surface and meibomian glands in patients with treatment-naive acne vulgaris.

**Methods:**

The Ocular Surface Disease Index (OSDI) questionnaire, invasive tear film breakup time, fluorescein staining of the ocular surface, and Schirmer II test were performed for all subjects. Total eyelid and meibomian gland secretion scores were assessed. Non-contact meibography was performed with the Sirius corneal topographic device.

**Results:**

The right eyes of 35 patients with acne vulgaris and 35 healthy volunteers were included the study. While the OSDI and staining scores were significantly higher in the acne group than in the control group (p=0.01 and p=0.003, respectively), the invasive tear film breakup time and Schirmer measurements were significantly lower in the acne group (p=0.000 and p=0.003, respectively). The total eyelid and meibomian gland secretion scores were also higher in the acne group than in the control group (p=0.003 and p=0.000, respectively). On the morphological evaluation of the meibomian glands, the thickening, thinning, tortuosity, and presence of ghost areas were statistically significantly more common in the acne vulgaris group than in the control group (p=0.000, p=0.001, p=0.05, and p=0.006, respectively). The percentage of the meibomian gland loss area was significantly high in the acne vulgaris group on both upper and lower meibography. The meibomian gland loss area positively correlated with total eyelid and meibomian gland secretion scores.

**Conclusion:**

Acne vulgaris may have a predisposition to meibomian gland dysfunction and ocular surface damage. Early recognition of meibomian gland and ocular surface alterations seems important, especially in acne vulgaris cases for which oral isotretinoin treatment is planned.

## Abstract (pt)

**Objetivo:**

Todos os indivíduos responderam ao questionário *Ocular Surface Disease Index* (OSDI) e tiveram avaliados o tempo de ruptura do filme lacrimal pelo método invasivo, a coloração da superfície ocular com fluoresceína e o teste de Schirmer II. Foram ainda avaliados o escore palpebral total e o de secreção das glândulas meibomianas. Foi realizada meibografia sem contato com um dispositivo topográfico corneano Sirius.

**Resultados:**

Foram incluídos no estudo os olhos direitos de 35 voluntários com acne vulgar e 35 saudáveis. Os escores do *Ocular Surface Disease Index* e da coloração foram significativamente maiores no grupo com acne em comparação com o grupo controle (p=0,01 e p=0,003, respectivamente), mas o tempo de ruptura do filme lacrimal pelo método invasivo e as medidas do teste de Schirmer II foram significativamente menores (p=0,000 e p=0,003, respectivamente). O escore palpebral total e o escore de secreção das glândulas meibomianas também foram maiores no grupo com acne que no grupo controle (p=0,003 e p=0,000). Na avaliação morfológica das glândulas meibomianas, o espessamento, o afinamento, a tortuosidade e a presença de áreas fantasmas nas glândulas foram mais comuns no grupo acne vulgar que no grupo controle, com significância estatística (p=0,000, p=0,001, p=0,05 e p=0,006 respectivamente). A porcentagem da área de perdas das glândulas meibomianas foi significativamente mais alta no grupo com acne vulgar, tanto na meibografia superior quanto na inferior. A área de perda das glândulas meibomianas demonstrou uma correlação positiva com o escore palpebral total e com o escore de secreção das glândulas meibomianas.

**Conclusão:**

A acne vulgar pode levar a uma predisposição para a disfunção das glândulas meibomianas e para danos na superfície ocular. Parece ser importante reconhecer precocemente as alterações das glândulas meibomianas e da superfície ocular, especialmente nos casos de acne vulgar para os quais se planeja o tratamento oral com isotretinoína.

## INTRODUCTİON

Acne vulgaris is a common chronic inflammatory skin disease characterized by open or closed comedones (blackheads and whiteheads) and inflammatory lesions, including papules, pustules, or nodules (also known as cysts)^(1)^. The mean onset of the disease is at the age of 11 years in females and 12 years in males, and the disease nearly affects 85% of adolescents and young adults^(2,3)^. Acne vulgaris is a disease of pilosebaceous units existing all over the skin except the soles and palms. The main pathogenic mechanism that plays an important role in the development of acne are follicular hyperkeratinization, microbial colonization with *Propionibacterium acnes,* sebum production, and complex inflammatory mechanisms involving both innate and acquired immunity^(1)^.

Meibomian glands are modified sebaceous glands in the lower and upper eyelid tarsus and secrete lipid-rich secretions called meibum, similar to the sebaceous glands secretions in skin. This meibum provides the stability of the tear film and prevents its evoporation. Non-contact meibographic techniques provide noninvasive imaging of meibomian glands morphology.

The pathogenetic mechanisms of acne can be expected to also affect the meibomian glands. Although oral isotretinoin therapy, which was used to treat acne vulgaris, has long been known to affect meibomian gland morphology and dry eye tests, the number of studies that evaluated the effect of acne vulgaris on meibomian glands in patients who do not receive treatment is limited in the literature^(4,5,6, 7)^. Therefore we aimed to investigate meibomian gland morphology with non-contact meibography and ocular surface alterations in patients with treatment-naive acne vulgaris.

## METHODS

### Study population and design

This prospective comparative study was approved by the ethics committee of our university and adhered to the tenets of the Declaration of Helsinki. The study was organized in accordance with the ethical standards set by the ethics committee of the Faculty of Medicine, Afyonkarahisar Health Sciences University. The study was conducted in the Department of Ophthalmology of Afyonkarahisar Health Sciences University. Informed consent was obtained from the patients and their legal guardians.

The acne vulgaris classifying or grading system is not standardized; however, acne is often categorized as mild, moderate, and severe in guidelines/recommendations and by clinicians treating patients ^(1,8,9)^. We used the 2016 European S3 Acne Guideline for grading (comedonal, mild-to-moderate papulopustular, severe papulopustular, moderate nodular, severe nodular, and conglobate acnes) and included patients with acne vulgaris, excluding those with only comedonal acne^(8)^. Patients who received systemic isotretinoin medication were excluded from the study. The age- and sex-matched control group consisted of patients without acne vulgaris who applied to the ophthalmology clinic. The exclusion criteria for both groups were as follows: contact lens wearing, history of ocular trauma or surgery, systemic disease except acne vulgaris, active conjunctivitis or allergy, and systemic or topical medications that can affect the ocular surface and meibomian glands.

The study group was comprised of 35 patients with acne vulgaris and 35 age- and sex-matched control patients. Only the right eyes were used for the analyses.

All the participants were evaluated with the Ocular Surface Disease Index (OSDI) questionnaire (Allergan, Irvine, CA, USA) before the examination to inquire subjective complaints related to the ocular surface^(10)^. The patients mark the frequency of their complaints in the 3 subscales. The final score was calculated and interpreted as follows: 0-12, normal; 13-22, mild dry eye disease; 23-32, moderate dry eye disease; and >33, severe dry eye disease^(11)^. The invasive tear film breakup time (TF-BUT) test was used for tear film stability after instillation of fluorescein. TF-BUT was measured 3 times, and its average was recorded in seconds. Conjunctival and corneal stainings were evaluated using the Oxford grading schema, which consisted of 5 panels (A-E). The staining score, ranging from 0 to 15, was calculated^(12)^. The basal tear film production was measured with the Schirmer II test (with anesthesia). After instillation of topical 0.5% proparacaine hydrochloride, standard Schirmer test strip (Whatman No. 41, 0.5´ 30 mm) was placed on the middle-outer third of the lower eyelid and the wetting was recorded in milimeters (mm).

Eyelid margins were examined for telangiectasies, lid margin irregularity, obstructed meibomian gland orifices, and anterior or posterior displacement of the mucocutaneous junction on slit-lamp biomicroscopy^(13)^. By evaluating each of them as 1 point, the eyelid score was calculated as 0 to 4. The meibum quality expressed by applying pressure to the middle part of the lower and upper eyelids with the index finger was examined. The expressed meibum scores were as follows: grade 0, clear; grade 1, cloudy; grade 2, cloudy with granular debris; and grade 3, thick and toothpaste-like^(14)^. For non-contact meibography Sirius (CSO, Florence, Italy), a corneal topographic device with the Phoenix-Meibography software was used. Images showing the meibomian gland structure were captured after everting of both the lower and upper eyelids. The device calculates the dropout area semi-automatically by percentage and calculates meiboscore as follows: grade 0, no loss at all; grade 1¼, ≤25%; grade 2, 26%-50%; grade 3, 51%-75%; and grade 4, >75%^(15)^. On meibograhy images, the morphological features of meibomian glands were also examined as reported by Ebenezer et al. (tortuous: at least one prominent tortuous configuration in the gland; shortened: the gland does not extend to its normal length; thinned: glands with a width less than half the width of a normal gland; thickened: glands have a width that is equal to or more than twice the width of a normal gland; fluffy areas: amorphous white substance in areas where normal glands should have been present; ghost areas: pale glands without a normal meibomian gland architecture; and dropout: an empty space where a gland should have been observed)^(16)^. Meibographic evaluations were performed blindly by the same experienced examiner.

### Statistical analysis

The IBM Statistical Package for the Social Sciences (version 24.0) software program was used for the data analysis. The analytical results of numerical data are shown as mean, standard deviation (SD), and minimum and maximum values. The analytical results of the categorical data are shown as frequency and percentage (%).The normality test results of the numerical variables were evaluated using the Shapiro-Wilk test. In addition, variables whose skewness and kurtosis values ranged from −1.5 to +1.5 and that showed normal distributions were accepted. The Student *T* test was used for normally distributed groups, and the Mann-Whitney *U* test was used for groups that did not have a normal distribution in the comparison of the means of two independent groups. The Pearson chi-square test and Fisher exact test were used in the analysis of the categorical variables. While evaluating the linear relationship of the continuous variables, the Pearson correlation coefficient was used in those with normal distributions, and the Spearman correlation coefficient was used in those with non-normal distributions. A p-value <0.05 was considered statistically significant.

## RESULTS

The study consisted of 35 eyes of 35 patients with acne vulgaris and 35 eyes of 35 healthy conrol subjects. Of the participants, 17 (48.6%) were female and 18 (51.4%) were male, with a mean age of 16.5 ± 2.6 years (range, 13-23 years) in both groups. The comparisons of the OSDI score, invasive TF-BUT, Oxford staining score, Schirmer II test score, meibomian gland (MG) secretion score, and total eyelid score are summarized in table 1.

**Table 1:** Comparison of ocular surface and eyelid parameters

	Acne group	Control group	p
OSDI score	20.46 ± 13.45 (0-43,2)	11.87 ± 7.78 (0-27,2)	0,01
Invasive TF-BUT (sec)	16.0 ± 2.9 (12-28)	18.2 ± 1.7 (15-22)	0.000
Oxford staining score	0.43 ± 0.85	0.03 ± 0.16	0.003
Schirmer II test (mm)	12.5 ± 1.5 (9-16)	13.6 ± 1.2 (11-16)	0.01
MG secretion score	0.60 ± 0.69 (0-2)	0.0	0.000
Total eyelid score	0.74 ± 1.09 (0-3)	0.11 ± 0.40 (0-2)	0.003
Telangiectasia (n, %)	8 (22.9%)	2 (5.7%)	0.04
Lid margin irregularity (n, %)	1 (2.9%)	-	1.0
Obstructed meibomian gland (n, %)	12 (34.3%)	2 (5.7%)	0.003
Displacement of the mucocutaneous junction (n, %)	5 (14.3%)	-	0.05

MG= Meibomian gland; OSDI= Ocular surface disease index; TF-BUT= tear film breakup time

While the OSDI and Oxford staining scores were significantly higher in the acne group than in the control group (p=0.01 and p=0.003, respectively), the invasive TF-BUT and Schirmer measurements were significantly lower in the acne group (p=0.000 and p=0.003, respectively). On eyelid examination, the presence of telangiectasia (p=0.04), obstructed MG (0.003), and displacement of the mucocutaneous junction (0.05) were significantly higher in the acne group. The groups shpwed no signiificant difference in terms of lid margin irregularity (p=1.0). The total eyelid and MG secretion scores were also higher in the acne group than in the control group (p=0.003 and p=0.000, respectively).

**Figure 1:**
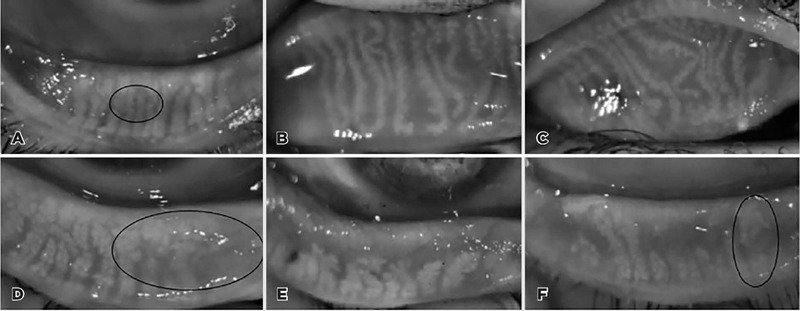
Non-contact meibography images of the upper (B, C) and lower eyelids (A, D–F) in the acne vulgaris cases: (A) thinning of the meibomian glands surrounded by a black circle; (B) tortuosity and thinning of meibomian glands and dropout area on the left side; (C) tortuosity in the meibomian glands greater than that depcited in image B; (D) ghost areas surrounded by a black circle; (E) marked meibomian gland loss area; and (F) thickening of a meibomian gland encircled with a black circle and meibomian gland loss.

Comparisons of the morphological features of meibomian glands, percentage of the MG loss area, and meiboscore obtained on non-contact meibography are shown in table 2. Thickening, thinning, tortuosity, and the presence of ghost areas on the MGs were statistically significantly more common in the acne vulgaris group than in the control group (p=0.000, p=0.001, p=0.05, and p=0.006, respectively) (Figure 1). No significant differences in the fluffy areas and MG shortening were found between the groups (p=0.07 and p=1.0, respectively). The percentage of the MG loss area and meiboscore were significantly high in the acne vulgaris group on both upper and lower meibography (for the MG loss area on upper and lower meibographies, p=0.004 and p=0.002, respectively; for the upper and lower meiboscores, p=0.006 and p=0.000, respectively).

**Table 2:** Comparison of the meibomian gland morphological features and loss areas

	Acne group	Control group	p
Morphological Features (n, %)			
Thickened	11 (31.4%)	-	0.000
Thinned	10 (31.4%)	-	0.001
Tortuosity	19(54.3%)	11 (31.4%)	0.05
Fluffy areas	10 (28.6%)	4 (11.4%)	0.07
Shortening	1 (2.9%)	-	1.0
Ghost areas	9 (25.7%)	1 (2.9%)	0.006
Upper meibography (%)	16.5 ± 9.7 (2.0-39.1)	10.8 ± 5.7 (1.2-22.3)	0.004
Upper meiboscore	1.2 ± 0.4	1.0 ± 0.0	0.006
Lower meibography (%)	19.6 ± 11.3 (2.3- 37.8)	12.6 ± 6.5 (2.1- 25.1)	0.002
Lower meiboscore	1.4 ± 0.5	1.0±0.0	0.000

In the acne group, a significant correlation was found between the total eyelid and MG secretion scores (r=0.64, p=0.000). The correlations of the total eyelid and MG secretion scores with the percentage of the MG loss area on meibography is given in table 3. The total eyelid and MG secretion scores significantly positively correlated with MG loss area in both the lower and upper eyelids.

**Table 3:** Correlations of the total eyelid and MG secretion scores with the MG loss area

	Upper meibography (%)		Lower meibography (%)	
	r	p	r	p
MG secretion score	0.50	0.002	0.50	0.002
Total eyelid score	0.67	0.000	0.70	0.000

## DISCUSSION

In the pathogenesis of acne vulgaris, which is a chronic inflammatory disease of the pilosebaceous unit, follicular colonization by *P. acnes*, sebum overproduction, abnormal shedding of the follicular epithelium, and inflammation are considered^(17,18,19)^. *P. acnes* is commensally present in normal skin flora and lives in a pilosebaceous unit, using lipid-rich sebum as a nutrient source, and produces a glycocalyx polymer, which causes to adherence to the walls of the follicular/sebaceous gland structure. This glycocalyx polymer acts as a biological glue that causes the plugging and adhesiveness of the pilosebaceous unit in acne vulgaris. In addition to the plugging effect, *P. acnes* can trigger inflammation by the activation of monocyte Toll-like receptor 2 and increasing the production of interleukin 12, which is a proinflammatory cytokine^(20)^. MGs are modified sebaceous glands and are inhabited by bacterial flora inherently. Anaerobic bacterial colonization has been shown to be more common in patients with meibomian gland dysfunction (MGD), and the dominant form is *P. acnes* isolated from both the MG secretion and conjunctival cul-de-sac. The same study also reported that flora is more complicated in patients with MGD^(21)^. Epithelial hyperkeratinization, which is involved in the pathogenesis of acne vulgaris, can cause plugging in the MGs and skin. Obstruction of the MG ducts may lead to bacterial colonization and deterioration of meibum secretions secondary to increaded meibum viscosity^(22,23)^. Circulating and cutaneously derived hormones especially androgens are also known to play a role in acne pathogenesis. MG secretions are also regulated by androgens, estrogens, progestins, retinoic acid, and growth factors, and possibly by neurotransmitters^(24)^. All these pathways can lead to MGD, including intraglandular cystic dilation, meibocyte atrophy, and gland dropout in patients with acne vulgaris.

The lipid-rich layer released by the MGs to the ocular surface prevents the evaporation of the tear film and provides tear film stability. As a result of hyperosmolarity and instability of the tear film, evaporative dry eye occurs. Inflammed and obstructed MGs are also strongly associated with ocular surface inflammation.

Systemic isotretinoin therapy, which is widely used in the treatment of nodulocystic acne vulgaris, has negative effects on the MG morphology and dry eye test results^(25)^. Düzgün et al. reported that MGs were decreased in size and density on meibography imaging after the administration of isotretinoin^(5)^. Even topical isotretinoin therapy has been reported to cause dry eye signs and symptoms^(26)^. Early recognition of MG and ocular surface alterations seems important, especially in acne vulgaris cases for which oral isotretinoin treatment is planned. The number of studies that evaluated patients with treatment-naive acne vulgaris is quite limited in the literature.

In this study, we found that while the OSDI (p=0.01) and Oxford ocular staining scores (p=0.003) significantly increased in the patients with acne vulgaris patients, their Schirmer measurements (p=0.01) and invasive tear film breakup times (p=0.000) decreased. The MG secretion (p=0.000) and total eyelid scores (p=0.003), including telangiectasias, obstructed MG orifices, and anterior or posterior displacement of the mucocutaneous junction, were also increased in patients with acne. In a study by Özdemir et al., including 50 nodulo-cystic acne cases, they showed that abnormal tear film breakup times and Schirmer scores were significantly more common in the acne group than in the control group^(7)^. The MG loss area and meiboscore were significantly higher in the acne group. Similarly to our finding, Muhafiz et al. claimed that tear breakup time was significantly lower (p< 0.001) and the loss rates in the MGs in both the upper (p=0.001) and lower eyelids (p<0.001) were greater in the acne group than in the control group^(27)^. On the morhological evaluation of the MGs, thickening, thinning, tortuosity, and presence of ghost areas were statistically significantly more common in the acne vulgaris group than in the control group (p=0.000, p=0.001, p=0.05, and p=0.006, respectively). The fluffy areas and MG shortening also showed no significant difference between the groups (p=0.07 and p=1.0, respectively). Although we cannot explain these morphological changes completely, we considered that obstruction of the meibomian glands secondary to sebum overproduction and epithelital hyperkeratinization may cause increased tortuosity and thickening of meibomian glands. As the pathological process continues, thinning and ghost areas may occur. Future large-scale and more-comprehensive studies are worthwhile for establishing the importance of these morphological changes and how they can be useful in clinical practice.

In addition, we observed that the total eyelid and MG secretion scores highly correlated with the percentage of MG loss area on both lower and upper meibography. Therefore, evaluation of the eyelids in acne vulgaris cases for which meibography cannot be performed provides valuable information about MGD.

The relatively small sample size and evaluation of the MG secretion score only on the lower eyelid are the limitations of this study. The main strength of this study include the functional evaluation with the OSDI questionnaire, morphological evaluation of the meibomian glands besides the quantitative assessment, and the use of the MG secretion score, which provides information about the quality of the meibum.

In summary, acne vulgaris leads to impairments in the functional and structural ocular surfaces. In addition, the total eyelid score, MG secretion score, and MG loss area were higher in the patients with acne. Meibomian gland thickening, thinning, and tortuosity, and presence of ghost areas were more common in the acne vulgaris group than in the control group. Acne vulgaris may have a predisposition to MGD and tear instability. Evaluation of the ocular surface and meibomian glands will be beneficial to patients with acne vulgaris, especially when systemic isotretinoin therapy is considered, and may be useful in deciding on the choice of treatment.
